# The effect of the type of foam pad used in the modified Clinical Test of Sensory Interaction and Balance (mCTSIB) on the accuracy in identifying older adults with fall history

**DOI:** 10.1142/S1013702520500134

**Published:** 2020-07-25

**Authors:** Rumpa Boonsinsukh, Bodin Khumnonchai, Vitoon Saengsirisuwan, Nithinun Chaikeeree

**Affiliations:** 1 Faculty of Physical Therapy, Srinakharinwirot University, Nakhon Nayok, Thailand; 2 Faculty of Physical Therapy and Sport Medicine, Rangsit University, Pathum Thani, Thailand; 3 Department of Physiology, Faculty of Science, Mahidol University, Bangkok, Thailand; nithinun@g.swu.ac.th

**Keywords:** Falls, older adult, postural stability, sensory integration

## Abstract

**Background:**

The type of foam pad used in the modified Clinical Test of Sensory Interaction and Balance (mCTSIB) influences the accuracy with which elderly fallers are identified. Two types of foam are commonly used in practice: Airex and Neurocom foam.

**Objective:**

The aim of this study was to assess the accuracy with which elderly fallers can be identified when the Airex foam and Neurocom foam are used in the mCTSIB.

**Methods:**

One hundred eighty-four elderly participants with a mean age of 69 years were classified into faller and nonfaller groups based on their 12-month fall history. Balance stability was measured under four conditions of the mCTSIB for 120 s each: standing on a floor or a foam pad with their eyes open or eyes closed. The time needed to maintain stability was measured by a stopwatch, and postural sway characteristics were measured using an acceleration-based system. Comparisons between groups were performed by two-way mixed ANOVA. The accuracy of differentiating elderly fallers from nonfallers with different foam types was evaluated using receiver operating characteristic curve (ROC) analysis. The time to maintain stability under four conditions of the mCTSIB (composite score) and under two conditions on the foam (foam score) were used for the ROC analysis.

**Results:**

The results showed that the nonfallers required more time to maintain stability and had a smaller sway area than the fallers (p<0.001). The foam led to a larger difference between groups, suggesting the use of foam in examining the risk of falls. The Airex and the Neurocom foam pads led to a large area under the curve (0.93 to 0.95) in identifying elderly fallers and nonfallers when the composite and foam scores were used. A cutoff score of 447/480 s for the composite score and 223/240 s for the foam score yielded a posttest accuracy of 88% to 89%, with a sensitivity of 0.80–0.92 and specificity of 0.88–0.95.

**Conclusion:**

In conclusion, Airex and Neurocom foam can be used interchangeably with guidance in the mCTSIB, as they led to the accurate identification of elderly fallers among older persons who could walk and live independently in the community.

## Introduction

Elderly persons frequently fall, and falls can result in minor or major life-threatening injuries that subsequently lead to a decline in their health-related quality of life.^1^ The ability to screen for elderly people at high risk of falls can guide the development of a suitable fall prevention program. Many factors lead to an increased risk of falls in elderly persons, including balance problems, environmental hazards (e.g., poor lighting and an unsafe walking surface),^2,3^ cognitive impairment,^4^ and the long-term usage of medications affecting balance.^5^ Among these factors, the impairment of the sensory systems used for balance is one of the important factors related to falls in elderly persons.^6,7^ Previous studies have shown that age-related declines in the function of the vestibular system,^8^ visual impairments,^6,9^ impaired somatic sensation,^7,10^ and impairment of the sensory systems used for balance were associated with a high risk of falls.^11,12^

A clinical test of sensory interaction and balance (CTSIB) was developed to assess the role of different sensory systems in balance control. The CTSIB consists of six balance conditions: standing on an unstable surface or a stable surface with eyes open, eyes closed, or a visual-conflict dome.^13^ Each condition lasts for 30 s and is repeated 3 times. An unstable surface is used to determine the ability of the central nervous system (CNS) to maintain stability when somatosensory input is an unreliable source of information. Standing with the eyes closed tests one’s ability to use proprioceptive or vestibular input for balance control. Wearing a visual-conflict dome tests one’s ability to select a reliable type of sensory input when the sensory information about the body’s position received from different sources is conflicting.^13^ The total duration required to maintain stability in all six conditions of the CTSIB has been used to identify elderly fallers (mean age of 80.5 years, SD=9.0), with a cutoff value of less than 260 s, a maximum duration of 540 s, a specificity of 0.90, and sensitivity of 0.44.^14^ This criterion of CTSIB for identifying elderly fallers is not practical in clinical practice, as it takes 9 min of quiet standing to obtain the results.^15^ Moreover, some test conditions in the CTSIB seem to be redundant; for example, the visual-conflict dome condition results were not different from those from the standing on unstable surface conditions.^16^

The modified CTSIB (mCTSIB) takes less time to administer than the original CTSIB due to the exclusion of 2 dome conditions, so it only lasts 4 min. The mCTSIB has been used to assess sensory interactions used for balance in adults (age range 22–83 years) with vestibular disorders^16^ and in patients with multiple sclerosis.^17^ The mCTSIB is preferable for use in the clinic due to its ease of use.^15^ All 4 conditions of the mCTSIB (standing on a floor or a foam pad with eyes open or with eyes closed) demonstrated excellent test–retest reliability (ICC=0.91 to 0.97)^18^ and moderate to high intrarater reliability (kappa=0.31 to 0.81).^19^ In order to identify fallers, a previous study showed that the measurement of the center of mass (CoM) acceleration during the mCTSIB test could identify fallers among patients with idiopathic Parkinson’s disease, suggesting that the mCTSIB was a valid clinical test to identify elderly people with neurological pathologies with a history of falls.^20^ In addition, scores on the sensory orientation test (e.g., standing on firm surface with eyes open, on foam and incline surfaces with eyes closed) demonstrated high accuracy in identifying elderly fallers.^12^ However, another study reported that the measurement of center of pressure sway speed during the mCTSIB test could not predict fall in community-dwelling elderly peoples who are active and independent.^21^ Therefore, it is a challenge to identify fallers among older people who are active and independent in the community using a simple clinical tool such as the mCTSIB.

The selection of a proper foam pad to be used in the mCTSIB test is also important for obtaining and interpreting accurate test results. A variety of foam types have been used among different studies of sensory interactions used for balance.^15,22,23^ Foam pads with different densities and Young’s modulus yielded different balance test results.^22,24^ Foam with proper density allowed the foam to comply with the body weight and trigger body sway in the appropriate amount, while foam with the highest Young’s modulus can be used to differentiate body sway between young and older adults better than foams with lower Young’s modulus.^22^ The Neurocom foam (Natus Incorporated, Inc.) and Airex foam (Airex AG, Inc.) have been widely used in clinical and laboratory settings.^20,22^ Both of them have yielded fair to good reliability (ICC (3,1)=0.41 to 0.81) in assessing balance with the mCTSIB test,^25^ but the costs of these foam pads are vastly different (the Airex foam is 160$USD, the Neurocom foam is 970$USD). A comparison of the foam pads’ clinical properties may provide information useful for selecting a cost-effective foam pad to screen for elderly fallers in the community. However, there is no study available at present that examines the accuracy of different foam types in identifying elderly fallers. Therefore, this study aimed to assess and compare the accuracy of using Airex and Neurocom foam pads to differentiate elderly fallers from nonfallers. Regarding to similar physical properties of Airex and Neurocom foam pads, we hypothesized that both types of foam would demonstrate similar accuracy in identifying older persons who had a history of falls.

## Methods

### Study design

This observational study was approved by the Human Research Protection Committee in Faculty of Physical Therapy, Srinakharinwirot University in Thailand (code: PTPT2017-010, approved date: 22 July 2017). Data collection was performed in the suburban communities in Pathum Thani Province in Thailand from July 2017 to July 2018.

### Participants

Male and female elderly individuals aged over 60 years, who could stand independently without assistive devices for at least 2 min, were able to walk independently with or without walking aids for at least 6 m and did not have a history of neurological diseases were recruited from suburban communities in Pathum Thani Province in Thailand. Individuals were excluded from this study if they met the following criteria: had signs or symptoms of vertigo, nystagmus, blindness, uncontrolled cardiovascular conditions, or neuropathy; had a severe musculoskeletal problem affecting balance performance; or were taking medications affecting balance (i.e., sedatives and hypnotics, antidepressants, and benzodiazepines).^5^ In addition, elderly individuals who had comprehension problems indicated by a score of less than 24 out of 30 on the Mini-Mental State Examination-Thai version 2002 (MMSE-Thai 2002)^26^ and had a body mass index (BMI) of equal to or higher than 30 kg ⋅ m${}^{-2}$ were excluded from this study. Informed consent was obtained from each participant before they participated in the study.

One hundred eighty-four was the target sample size of elderly persons for this study. The mean and standard deviation of the time to maintain balance with the mCTSIB test that were reported in a previous study^14^ were used to calculate the sample size. The participants were classified into non-fallers and fallers (i.e., persons who had fall at least once) based on their 12-month fall history. In this study, a fall was defined as coming to rest inadvertently on the ground or other lower level, excluding intentional change in position to rest in furniture, wall or other objects.^27^ To meet the target sample size, 325 elderly persons were recruited. Sixty-four persons were excluded from the study because they had neurological diseases (19 persons), had severe musculoskeletal problems (28 persons), were taking medications affecting balance (17 persons), or had personal issues that were not related to health problems (77 persons).

## Procedures

The participants were interviewed about their medical history, current medication use, 12-month fall history, ability to walk, and use of a walking aid. Manual muscle tests for the hip, knee, and ankle muscles were performed to characterize lower limb muscle strength. The fear of falling of each participant was determined using the falls efficacy scale (FES). The total score of the FES ranges from 0 to 100. A higher FES score indicates lower confidence in performing daily activities and a higher fear of falling.^28^

Two types of foam pads, the Neurocom and Airex foam pads, were used in the foam conditions in the mCTSIB. The dimensions of the Neurocom and Airex foam pads were 0.46×0.46 m and 0.50×0.41 m, respectively. Two pieces of the Airex foam pads were stacked to ensure that the two types of foam had similar thicknesses (0.12 m for the Airex and 0.13 m for the Neurocom foam). Volume and mass of the foam pad were used to calculate density. Densities of a stacked of two-pieces Airex foams and one piece of the Neurocom foam had density of 55 kg ⋅ m${}^{-3}$ and 60 kg ⋅ m${}^{-3}$, respectively.

A fabric cover was used to cover each type of foam pad to blind the participants to the foam type. Prior to the data collection, an examiner (BK) demonstrated the test, and the participants were allowed to practice the test until they became familiar with each test condition. All the participants underwent the four conditions of the mCTSIB, including standing on the floor or on foam with their eyes open or eyes closed. Our pilot study showed that elderly participants who met our inclusion and exclusion criteria could stand for more than 30 s but not longer than 120 s. Therefore, the allowed duration for each condition was extended from 30 s, as indicated in the original protocol, to 120 s.^16^ The participants were asked to maintain stability in a standing posture: standing barefoot with their feet shoulder width apart and arms crossed, touching their shoulders for as long as possible, for up to 120 s. Each condition of the mCTSIB test was performed once, resulting in a total of 6 conditions: (1) eyes open and standing on the floor, (2) eyes closed and standing on the floor, (3) eyes open and standing on the Neurocom foam pad, (4) eyes closed and standing on the Neurocom foam pad, (5) eyes open and standing on the Airex foam pad, and (6) eyes closed and standing on the Airex foam pad. Each participant performed a total of six trials in a random order using computer-generated randomization. A few minutes of rest between trials was allowed, in which the participated sat, stretched their lower limb muscles and walked a few meters to prevent fatigue. To ensure participant safety, vital signs and blood pressure were monitored prior to and after the test, and closed guarding during the tests was administered. The test was discontinued if any of the following criteria were met: the participant left the study before completion or had signs and symptoms such as dizziness, confusion, vomiting, angina or chest pain at rest, a blood pressure higher than 160/100 mmHg at rest, or a heart rate of more than 100 bpm at rest. The test was stopped immediately if needed. The total testing duration for each participant was approximately 40 min. According to those criteria, none of participants was discontinued from the test.

The time required to maintain stability in the standing posture and postural sway characteristics of the participants were measured during the mCTSIB. A stopwatch was used to record the time from when a researcher (BK) said “start” to when the participant could not maintain the starting position. A trial was terminated if the participant’s feet or arms changed positions, his or her eyes opened during the eyes closed condition, he or she required manual assistance to prevent a fall,^15^ or the maximum time limit was reached. The measurement of postural sway characteristics was used with an acceleration-based equipment and ISway software plug-in (APDM, Mobility Lab^TM^).^29^ Prior to each testing condition, an OPAL inertial sensor strapped at the fifth lumbar vertebra (L5). The acceleration signals were collected at a sampling rate of 50 Hz for further processing by the software plug-in. The evaluation was performed in the same setting, and all participants received the same verbal instructions. Our pilot study was performed in 10 elderly persons and the reliability of the measurement was analyzed using an intraclass correlation coefficient, ICC (2,1). The pilot results showed an excellent intrarater reliability of a stopwatch measurement (ICC=0.998, 95% CI=0.996–0.999, p<0.001) and the inertial sensor-based assessment (ICC=0.999, 95% CI=0.996–0.999, p<0.001) of time required to maintain stability.

## Data Analysis

**Fig. 1.**
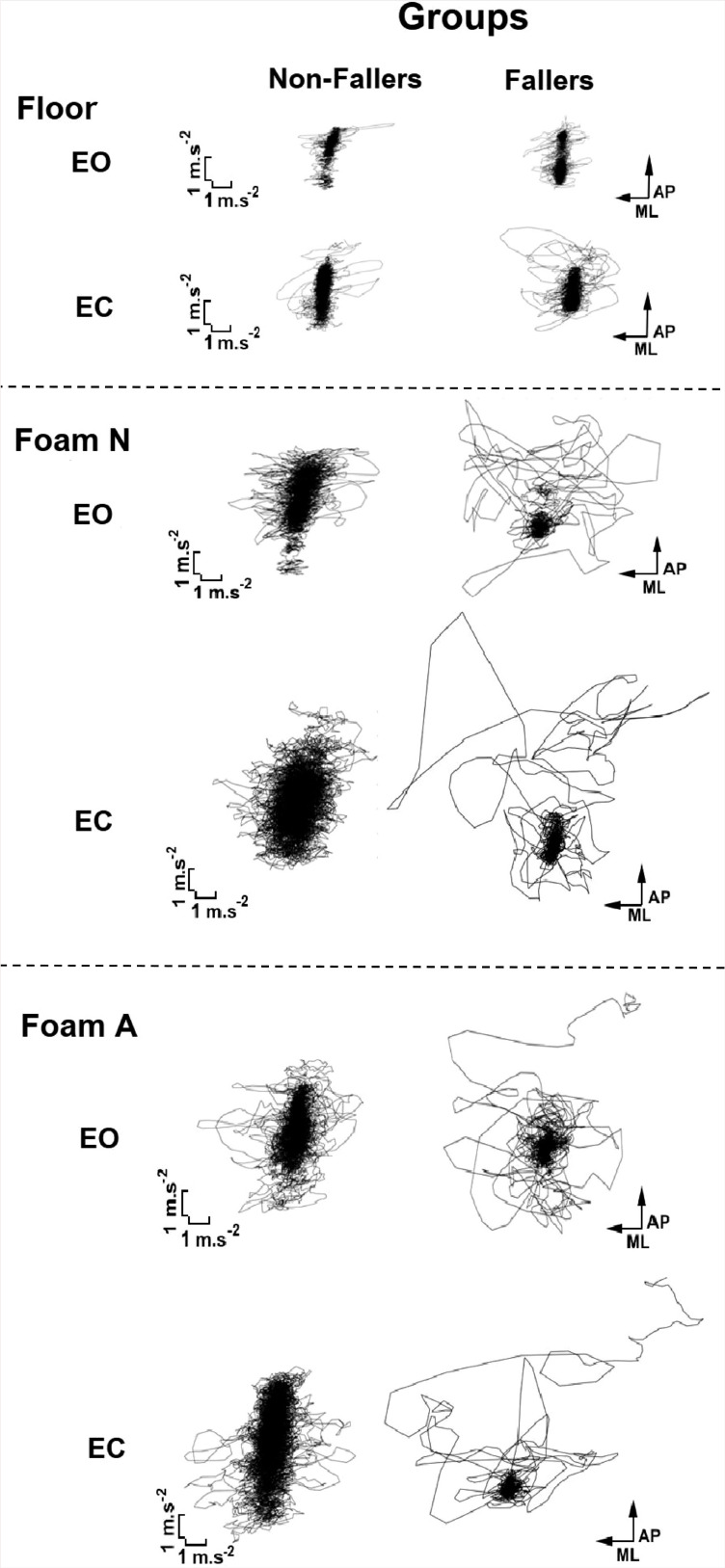
The raw trace of center of mass acceleration in the horizontal plane for two representative participants in the mCTSIB using the Neurocom foam (Foam N) and the Airex foam (Foam A). AP: anteroposterior, ML: mediolateral.

The demographic and clinical characteristics of the participants were assessed using descriptive statistics. The baseline characteristics between the fallers and nonfallers were examined using a two-sample t-test. The mean and standard deviation of the time required to maintain stability and postural sway for each group were estimated for each test condition. The software plug-in (APDM, Mobility Lab^TM^) contained algorithms for estimation of the CoM position and automatically computed postural sway measures. The area enclosed by the acceleration path in the medial-lateral plane (sway area) (m^2^ ⋅ s${}^{-4}$) and root mean square of acceleration (m ⋅ s${}^{-2}$) in the anteroposterior (RMS-AP) and mediolateral directions (RMS-ML) were used to characterize the postural sway of the participants. The raw trace of the CoM acceleration in the horizontal plane during the test were plotted using a software, MALTAB^TM^ and representatives of a faller and a nonfaller participant in the mCTSIB using the Neurocom foam and the Airex foam were selected (Fig. 1). The effect of testing conditions (4 conditions) and groups (faller and non-faller) of each foam on balance performance was examined using a 2-way mixed ANOVA. Interaction effect of conditions and groups was analyzed to determine whether the testing condition influenced balance performance differently between faller and nonfallers. Bonferroni correction was used as *post hoc* tests. Statistical significance was set at a p-value of less than 0.05. The analysis was performed with IBM SPSS${}^{\text{®}}$ Statistics, version 25 (ICN: 7937000).

The amount of time a participant could stand in each test condition was used to calculate the total time required to stay in the standing posture. Two methods for calculating the total time were used. The first method involved the calculation of a composite score, which was defined as the sum of the time required to maintain stability under the four conditions of the mCTSIB test; the maximum total score was 480 s (4 conditions × 120 s per condition). The second method involved the calculation of the foam score, which as defined as the sum of the time required to maintain stability when standing on foam in the foam conditions. The total possible score for the foam score was 240 s (2 conditions × 120 s per condition). The composite and foam scores were compared between 2 foam types and 2 groups of elderly persons using two-way mixed ANOVA. The level of statistical significance was set at a p-value of less than 0.05.

The accuracy of the foam used in the balance test in differentiating fallers and nonfallers was analyzed using receiver operating characteristic (ROC) analysis.^30^ The cutoff points of the foam score that maximized the sensitivity and specificity were chosen. Likelihood ratios were calculated to determine the strength of the foam test.

## Results

One hundred eighty-four elderly persons, participated in this study, and 92 persons were in each of the two groups (fallers and nonfallers). None of them were taking medications affecting balance. The participants’ demographics, clinical characteristics, and fall histories are summarized in Table 1. The faller and the nonfaller groups had similar demographic and clinical characteristics (p>0.050), except for the FES scores (p=0.010), where the faller group showed significantly higher scores (Table 1).

**Table 1.** Participants’ demographic and clinical characteristics.

Demographic and characteristics		Nonfallers (n=92)	Fallers (n=92)	P-value
Age (years)		68.2 ± 5.6	69.5 ± 5.8	0.109
Weight (kg)		66.1 ± 6.1	67.87 ± 6.3	0.055
Height (cm)		166.28 ± 5.9	165.35 ± 5.5	0.271
BMI (kg/m^2^)		23.81 ± 2.2	24.75 ± 2.6	0.074
Sex (%)	Female	55.4	58.7	
	Male	44.6	41.3	
Number of falls (%)	0	100.0	0.0	
	1	0.0	38.0	
	More than 1	0.0	61.9	
Fall locations (%)	Indoor	0.0	42.4	
	Outdoor	0.0	57.6	
Types of falls (%)	Slip	0.0	30.4	
	Trip	0.0	47.8	
	Postural transition	0.0	21.7	
Taking medications (%)	Antihypertensive drugs	13.0	7.6	
	DM medications	7.6	8.6	
Walking aids (%)	Cane	4.3	17.3	
	Walker	2.1	5.4	
LE muscle strength (/5)		4	4	—
FES score		67.5 ± 6.8	94.7 ± 5.5	0.001*
MMSE-Thai 2002 (/30):		27.1 ± 1.9	26.6 ± 1.9	0.096

*Notes*: Values are shown as the mean ± SD, except the lower extremity (LE) muscle strength is presented as the median. LE strength refers to the hip, knee, and ankle muscle strength. kg=kilogram; cm=centimeter; BMI=Body mass index; bpm=beats per minute; MMSE-Thai 2002=The Mini Mental State Examination-Thai version 2002. * = significant difference between fallers and nonfallers at *p* < 0.05.

**Fig. 2.**
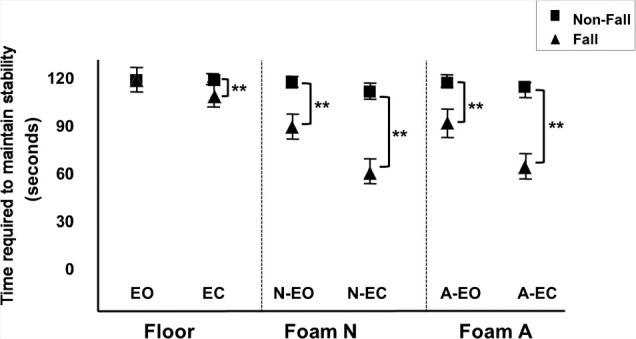
Comparison of the time required to maintain stability in each condition between the fallers and nonfallers. Values are shown as the mean ± SD, EO; floor - eyes opened, EC; floor - eyes closed, N-EO; Neurocom foam - eyes opened, N-EC; Neurocom foam eyes closed, A-EO; Airex foam - eyes opened and A-EC; Airex foam eyes closed. **Significant difference between fallers and nonfallers at p<0.001.

**Fig. 3.**
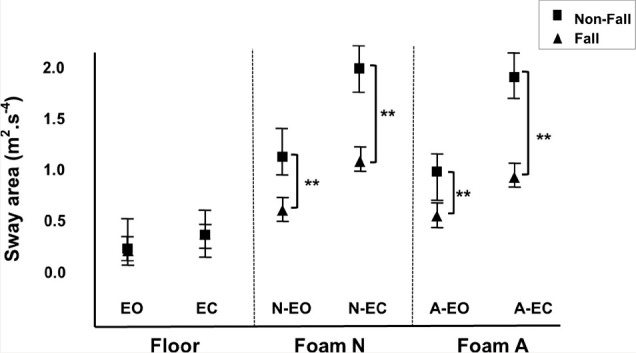
Mean sway area in the mCTSIB conditions between faller and nonfaller groups when using the Neurocom and Airex foam. Values are shown as the mean ± SD, EO; floor - eyes opened, EC; floor - eyes closed, N-EO; Neurocom foam - eyes opened, N-EC; Neurocom foam eyes closed, A-EO; Airex foam - eyes opened and A-EC; Airex foam eyes closed. **Significant difference between fallers and nonfallers at p<0.001.

Time to maintain stability was significantly affected by groups (fallers and nonfallers) ($F_{\left(1,182\right)}=160.05$, p<0.001) and conditions, six combinations of two visual conditions and three surface conditions ($F_{\left(2.57,467.53\right)}=55.89$, p<0.001). In addition, this parameter was significantly affected by an interaction effect of conditions and groups ($F_{\left(2.57,467.53\right)}=8.62$, p<0.001). The differences in the time required to maintain stability between the faller and nonfaller groups were evident during all testing conditions (p<0.001), except the eyes open while standing on the floor condition (p=0.620) (Fig. 2). The major differences in the time required to maintain stability between the faller and nonfaller groups were found between the conditions in which the participants stood on the foam (Fig. 2). These findings were similar for the Neurocom and Airex foam conditions (Fig. 2). The CoM acceleration trajectories during standing in the mCTSIB conditions for a representative faller and nonfaller are shown in Fig. 1. The magnitudes of acceleration were larger and more dispersed in the fallers compared to the nonfallers for all mCTSIB conditions and all types of foam, except when the participants stood on the floor (Fig. 1). The sway area was significantly affected by groups ($F_{\left(1,182\right)}=15.65$, p<0.001) and conditions ($F_{\left(2.57,410.07\right)}=184.96$, p<0.001). In addition, this parameter was significantly affected by an interaction effect of conditions and groups ($F_{\left(2.57,410.07\right)}=115.54$, p<0.001). Significant differences in the sway area between groups were found when the participants stood on the foam (p<0.001) but not when they stood on the floor (p=0.615 to 0.862) (Fig. 3). In addition, the RMS-AP and the RMS-ML of the CoM acceleration were different between the faller and nonfallers (p<0.001).

The mean and standard deviation of the composite and foam scores are shown in Table 2. There was a significant effect of group (p<0.001) without an interaction effect (p=0.053), indicating that the faller group had a significantly lower composite score than the nonfaller group (p<0.001) when either the Neurocom foam or Airex foam was used in the mCTSIB (p<0.001). Consistent results were found for the foam scores; the faller group had significantly lower foam scores than the nonfaller group when using either foam (p<0.001). The composite and foam scores may be used to reveal differences in balance ability between the faller group and the nonfaller group. Therefore, both scores were used in the receiver operating curve (ROC) analysis.

The Neurocom and the Airex foam yielded high accuracy in differentiating fallers from nonfallers, with a large area under the ROC curve (0.93 to 0.95) when using the composite score and foam score. High sensitivity (0.80 to 0.92) and high specificity (0.88 to 0.95) were obtained at a cutoff score of 447 out of 480 s and 223 out of 240 s for the composite score and the foam score, respectively (Table 3).

**Table 2.** Means and standard deviations of composite and foam scores in the faller and nonfaller groups.

	Fallers (n=92)		Nonfallers (n=92)	
Types of score	Mean ± SD	Range	Mean ± SD	Range
Composite score (/480 s)				
Neurocom	382 ± 64	220–470	471 ± 25*	325–480
Airex	388 ± 63	223–471	474 ± 21*	332–480
Foam score (/240 s)				
Neurocom	152 ± 54	15–230	231 ± 25*	85–240
Airex	159 ± 53	18–231	234 ± 21*	92–240

*Notes*: *Significant difference in the composite scores and foam scores between the fallers and nonfallers at p<0.001. N: number of participants, SD: standard deviation.

**Table 3.** Area under the curve, cutoff score, sensitivity, specificity, likelihood ratio and percentage accuracy of foam score.

	Composite score		Foam score	
Measures	NeuroCom	Airex	NeuroCom	Airex
AUC (95% CI)	0.94 (0.90–0.97)	0.95 (0.92–0.98)	0.93 (0.90–0.97)	0.95 (0.92–0.98)
Cutoff score	447/480	447/480	223/240	223/240
Sensitivity (95% CI)	0.89 (0.81–0.93)	0.80 (0.80–0.81)	0.92 (0.87–0.98)	0.90 (0.86–0.98)
Specificity (95% CI)	0.89 (0.83–0.95)	0.95 (0.94–0.95)	0.88 (0.81–0.95)	0.92 (0.82–0.95)
Positive LH	8.17	14.18	7.73	11.86
Negative LH	0.12	0.21	0.09	0.11
Posttest accuracy (%)	89.13	88.00	90.00	91.00

*Notes*: AUC: area under the curve, CI: confidence interval, posttest accuracy was calculated using the selected cutoff score, LH: likelihood ratio.

## Discussion

The aim of this study was to compare the accuracy of using Airex and Neurocom foam pads to differentiate elderly fallers from nonfallers. The results demonstrated that both Airex and Neurocom foam are accurate in identifying elderly persons with a history of falls. Our findings also supported the use of foam in examining the risk of falls because the foam conditions showed larger differences in balance control between fallers and nonfallers than the floor conditions. The between-group differences in the time required to maintain stability and sway characteristics that were evident only in the foam conditions were similar to those reported in previous studies in which different methods (e.g., center of pressure balance assessment) were used to determine the duration required to maintain balance and postural sway.^11,31,32^ The similarity of the clinical properties (time required to maintain stability and sway characteristics) between the Airex foam and the Neurocom foam may be explained by the similarity in their densities (55 kg ⋅ m${}^{-3}$ and 60 kg ⋅ m${}^{-3}$, respectively), Young’s modulus elasticity values (0.26 and 0.14 megapascal, respectively), and their size.^22,25^ These properties cause the foam to be an unstable support surface and trigger an individual to use his or her vestibular system to control his or her balance.^24^ Because the mechanical properties of the two types of foam are similar, the balance control system of a participant is challenged to the same extent when he or she stands on either type of foam.

In addition to density and Young’ s modulus, thickness of foam pad (a stack of 2 Airex foams) may cause the Airex foam in this study to produce large postural sway and reveal similar test results as compared to the Neurocom foam. A previous study in healthy adults reported that one Airex foam could not differentiate postural sway between standing on floor and on foam, while a blue latex pad and an air-filled circular pad could.^33^ The thickness of one Airex foam may cause the foam to become a floor-like surface, thus reducing the ability to produce unstable surface. Another study focusses on the effect of foam on postural sway in participants of different weights.^34^ One-piece Airex foam produced largest sway in persons with less than 90-kilogram body weight,^34^ suggesting that one piece of this foam had low ability to produce sway when it was used in a person with heavy weight. In contrast, our current study used a stack of two-piece Airex foam pads (0.12 m thickness), thus, allowing the Airex foam to appropriately produce unstable condition for assessing balance and screening falls in a comparable manner to the Neurocom foam.

The cutoff scores for the Neurocom and the Airex foam in the mCTSIB provided high sensitivity and specificity when the composite score and foam score were used in the ROC analysis. The high positive likelihood ratio, which was greater than 5, and the low negative likelihood ratio, which was not more than 0.2, confirms that the strength of the test is very good.^35^ These results indicated a high probability of identifying elderly fallers and a high probability that elderly nonfallers will screen negative when these foam pads are used in the standing balance test. The cutoff scores presented in this study (447 out of 480 s for the composite score and 223 out of 240 s for the foam score) differed from the score reported in a previous study (260 out of 540 s).^14^ However, a specificity of over 90% for identifying fallers was reported in both studies.^14^ The discrepancy in the cutoff scores between the present and the previous study is due to differences in the number of conditions, the repetitions of the test performed, and the duration of the trial performed for each condition. In the previous study, three repetitions of the six conditions of the CTSIB (30 s each condition) were administered, whereas the present study allowed no repetitions of the foam conditions in the mCTSIB, and each condition lasted for 120 s. This study demonstrated that the mCTSIB test was accurately identify fallers, while the previous study found that the mCTSIB failed to predict fall in community-dwelling elderly people.^21^ Disagreement in findings in both studies could be due to the differences in testing duration. As oppose to 2-min trial in current study, the 20-s trial used in the previous study may not be sufficient to differentiate balance ability between elderly who prone and not prone to fall in those who were active elderly.

Time constraints and the ability of the participants can be used to guide the selection of either the composite score or the foam score in practice. To use the composite score, 8 min of testing are required to complete the four test conditions (standing on the floor or on foam with two visual conditions). Not only is standing quietly for 8 min time consuming, but it can also lead to physical fatigue. However, the conditions in which the participant stands on a floor with eyes open or eyes closed can be used to ensure individuals’ safety. Fear of falling^36^ and visual dependence^37^ in elderly people can generally increase the risk of an individual losing his or her balance. This characteristic was also observed in the present study, where the elderly fallers had higher FES scores than the nonfallers did. Therefore, starting the test with the standing on a floor condition, which is a less challenging task, may help to reduce the participant’s fear of falling when performing the mCTSIB, especially for those who may not feel comfortable with their eyes closed. In contrast, it takes less time (4 min) to calculate the foam score, as only the conditions in which the participant stands on the foam with eyes open or eyes closed are performed. However, to use the foam score, the therapist should ensure that the participants are able to stand independently with their eyes open and eyes closed prior to asking them to stand on the foam. Moreover, the foam pad used in this study was covered with the fabric cover. Different materials of fabric cover may influence the testing difficulty and hence this aspect needs to be addressed further in the future study.

This study has limitations. The fall-related information was acquired retrospectively; thus, this information may be subjected to recall bias. However, the fact that our participants were able to provide details on their falls (locations and types of falls) indicated that their fall history information was fairly reliable. The generalization of the results is limited to elderly persons who have no neurological diseases and are able to walk independently with or without walking aids over at least for a short distance (6 m). Future studies should also be carried out in frail elderly individuals. In this study, although a single testing for each condition of mCTSIB was administered, participants were allowed to practice until they are familiar with the testing condition. This could also be a limitation in the clinical practice where there was not sufficient time for assessment. However, conducting an additional trial of balance tests and using the average of several trials would be better for reliability of outcome assessment. Having a foam pad that is more accessible and less expensive than the foam pads used in this study may increase the likelihood that this screening test is used in community practices. Therefore, a foam pad needs to be developed domestically, and additional studies need to be conducted to evaluate its clinical properties.

## Conclusion

In conclusion, Airex foam and Neurocom foam can be used interchangeably with strict guidance when administering the mCTSIB test to identify elderly individuals who have a history of falls. Preparation of the equipment (two pieces of the Airex foam with fabric cover), assessment procedure, and the selection of scoring methods should be considered for clinical application of this test. Cutoff scores from 2 methods of analysis, including the total time required for the four conditions of the mCTSIB (composite score) and the total time required for only two foam conditions (foam score), can be used to identify elderly fallers with high accuracy. The cutoff values are 223/240 s for the composite score and 447/480 s for the foam score.

## Conflict of Interest

There were no conflicts of interest. No authors have financial interest in any of the research materials or equipment used in this study.

## Author Contributions

Conception and design of study: Rumpa Boonsinsukh; acquisition of data: Bodin Khumnonchai; analysis and/or interpretation of data: Nithinun Chaikeeree, Bodin Khumnonchai. Drafting the manuscript: Bodin Khumnonchai, Nithinun Chaikeeree; revising the manuscript critically for important intellectual content: Rumpa Boonsinsukh, Nithinun Chaikeeree.

All authors approved the version of the article to be published.

## Acknowledgments

This study was funded in part with the integrated thesis & research management system (iThesis) fund from the Graduate School of Srinakharinwirot University. The authors are grateful to the participants who volunteered for this study, to the staff at Tambon Lak Hok Health Promotion Hospital for the recruitment and monitoring of the elderly cohort, and to Faculty of Physical therapy, Rangsit University for access to the clinical setting and equipment.
